# Social group and health care provider interventions to increase the demand for malaria rapid diagnostic test among community members in Ebonyi state, Nigeria: study protocol for a cluster randomized controlled trial

**DOI:** 10.1186/s13063-019-3620-0

**Published:** 2019-10-10

**Authors:** Ugwu I. Omale, Benedict N. Azuogu, Chihurumnanya Alo, Ugochukwu C. Madubueze, Onyinyechukwu U. Oka, Kingsley C. Okeke, Ifeyinwa M. Okafor, Rowland Utulu, Uduak E. Akpan, Chijioke V. Iloke, Anthonia O. Nnubia, Ifeyinwa I. Eze, Ogechukwu C. Anene, Chukwuka R. Nnabu, Deborah C. Ibemesi

**Affiliations:** 1 Department of Community Medicine, Alex Ekwueme Federal University Teaching Hospital, Abakaliki (AEFUTHA), Abakaliki, Ebonyi state Nigeria; 2 Department of Community Medicine, Ebonyi State University (EBSU), Abakaliki, Ebonyi state Nigeria; 3 Department of Community Medicine, Alex-Ekwueme Federal University Ndufu-Alike (AE-FUNAI), Abakaliki, Ebonyi state Nigeria; 4 Nigerian Field Epidemiology and Laboratory Training Programme (NFELTP), 50 Haile Selassie Street, Asokoro, Abuja Nigeria; 5 Government House Clinic, Abakaliki, Ebonyi state Nigeria

**Keywords:** MRDT, Fever, Malaria, Demand, Social groups, Providers, Community members

## Abstract

**Background:**

The World Health Organization recommended (in 2010) universal testing for suspected malaria, due to some fundamental changes in malaria trends such as the declining incidence of malaria in high-burden countries, the emergence of parasite resistance to anti-malarial drugs especially artemisinin-based combination therapies (ACTs) and the increased availability of diagnostic testing such as the malaria rapid diagnostic test (MRDT). The Nigerian government has long adopted this recommendation and with the support of foreign partners has scaled up the availability of MRDT. However, the malaria/MRDT rate in the communities is still far short of the recommendation. This study aims to evaluate the effectiveness of social group and social group/provider interventions in increasing the demand (use and/or request) for MRDT among community members with fever or malaria-like illness in Ebonyi state, Nigeria.

**Methods:**

A three-arm, parallel, stratified cluster randomized design will be used to evaluate the effect of two interventions compared to control: control involves the usual practice of provision of MRDT services by public primary healthcare providers and patent medicine vendors; social group intervention involves the sensitization/education of social groups about MRDT; social group/provider intervention involves social group treatment plus the training of healthcare providers in health communication about MRDT with clients. The primary outcome is the proportion of children under 5 years of age with fever/malaria-like illness, in the 2 weeks preceding a household survey, who received MRDT. The co-primary outcome is the proportion of children ages 5 years and above and adults (excluding pregnant women) with fever/malaria-like illness, in the 2 weeks preceding a household survey, who received MRDT. The primary outcome will be assessed through household surveys at baseline and at the end of the study.

**Discussion:**

The pragmatic and behavioural nature of the interventions delivered to groups of individuals and the need to minimize contamination informed the use of a cluster-randomized design in this study in investigating whether the social group and social group/provider interventions will increase the demand for MRDT among community members. “Pragmatic” means the interventions would occur in natural settings or real- life situations.

**Trial registration:**

ISRCTN, ISRCTN14046444. Registered on 14 August 2018.

**Electronic supplementary material:**

The online version of this article (10.1186/s13063-019-3620-0) contains supplementary material, which is available to authorized users.

## Background

The World Health Organization (WHO) recommended in 2010 that all patients suspected of having malaria receive prompt parasitological testing (with microscopy or malaria rapid diagnostic test (MRDT)) to confirm diagnosis before treatment [1]. Treatment based on presumptive diagnosis should only be considered when parasitological diagnosis is not accessible [1]. However, patients suspected of having severe malaria, including children and high-risk groups, should receive immediate treatment on presumptive grounds when the parasitological diagnostic test result is delayed by up to 2 hours or more [1]. The WHO recommendation for universal testing is based on some fundamental changes in malaria trends worldwide such as the declining incidence of malaria in high-burden countries, the emergence of parasite resistance to anti-malarial drugs especially to artemisinin-based combination therapies (ACTs) and the increased availability of diagnostic testing such as MRDT [2, 3]. Though malarial parasite resistance to ACTs has been detected in the Greater Mekong Area [4–7], ACTs are still effective because the parasite resistance is either in the form of delayed parasite clearance or resistance to the partner drug [4, 6, 7]. However, widespread over-treatment of malaria with ACTs as a result of inaccurate presumptive diagnosis is likely to compound the problem.

The use of MRDT is a key part of the strategy for universal parasitological diagnostic testing recommended by the WHO [1, 2, 8, 9] and this has been demonstrated in some countries like Senegal [10, 11] and Lao People’s Democratic Republic [10]. Many countries have started emphasizing parasitological diagnostic testing as the basis for malaria treatment and have been scaling up the availability and use of MRDT, especially in the public sector. It is worth noting that uptake and use of MRDT can only remedy over-treatment with ACTs when providers (and patients) respond appropriately to negative test results [12]. Accurate diagnosis of malaria is important in all settings [1, 13]: in settings where malaria is endemic, highly sensitive diagnosis is essential, especially in children in whom falciparum malaria can quickly become fatal; in all settings, highly specific diagnosis will minimize unnecessary anti-malarial treatment and improve the diagnosis of other causes of fever. The WHO recommended a policy of “test, treat and track” in 2012 to improve the quality of care and surveillance [3].

Malaria is a disease of public health importance in Nigeria. It is endemic, with varying endemicity and risk across the regions of the country [14–16]. Nigeria also bears about 29% of the malaria burden in Africa [15] and more than 25% of global malaria cases and of global mortality from malaria in 2015 [4]. Malaria rapid diagnostic test (MRDT) was introduced in Nigeria during the period of the national malaria strategic plan 2009–2013, with the aim of extending MRDT to all public and private sector health facilities including at the community level [14, 17, 18]. Aligning with the WHO’s Global Technical Strategy (GTS) for Malaria 2016–2030, the current national malaria strategic plan 2014–2020 aims for Nigeria to achieve a pre-elimination status and reduce malaria mortality to zero by 2020 [14]. One of the objectives of the 2014–2020 national malaria strategic plan is to, by 2020, perform MRDT or microscopy for all patients with suspected malaria who seek medical care, and the strategic actions include to ensure the availability of and access to MRDT (and or microscopy) at public and private health facilities; to build the capacity of healthcare personnel and to create demand for the utilization of parasitological diagnostic testing via actions targeted at both the health workers and the general public [14].

The WHO’s recommendation of universal parasitological testing has long been adopted by countries around the world, including Nigeria. The Nigerian government (with the support of foreign partners) has scaled up the availability of MRDT in recent years but significant challenges remain. The malaria/MRDT rate in the communities is still unacceptably low. In the recent Nigeria Malaria Indicator Survey (NMIS) 2015 [15], the percentage of febrile children under 5 years of age who (in the preceding 2 weeks) were reported to have had blood taken from a finger or heel for testing (indicating a malaria test) was 13% nationally and 11% in the south-east, indicating a very low test rate in the south-east zone, which includes Ebonyi state. Also, the World Malaria Report 2016 [19] reported that among 22 nationally representative surveys in sub-Saharan Africa between 2013 and 2015, the median percentage of febrile children who received a finger or a heel stick, indicating that a malaria diagnostic test was performed, was 51% in the public sector, 40% in the formal private sector and 9% in the informal private sector (which includes the patent medicine vendors (PMVs)).

Also, the scale-up has mainly been in the public health sector (and only to a lesser extent in the private sector) despite the fact that in Nigeria the majority of febrile patients (adults and children), especially from the less educated, lower socio-economic groups and in the rural settings, first seek care in the informal private sector (from the PMVs) [15, 20–22] where they are more likely to receive inappropriate treatment for malaria [15, 20–24], usually based on presumptive diagnosis [22–24]. This problem is similar across sub-Saharan African countries [4, 19, 25–27].

Moreover, following the recommendations in 2006 [28] for the use of ACTs as the first-line anti-malarial drug, there has been a progressive and widespread increase in the use of ACTs across Nigeria [15, 20, 29] and sub-Saharan African countries [4, 19] that is associated with over-treatment (over-prescription). This has heightened the risk of selection pressure and drug resistance. Though there is an increased rate of testing in the public facilities (which is still below recommendation), the test rate in the private sector, especially the informal private sector such as the PMVs, is at best negligible and the problem of non-adherence to negative test results is limiting the remedy to over-prescription of ACTs by MRDT [12].

The demands of patients and caregivers significantly influence the treatment decisions of the providers in general [30–33] and following (negative) MRDT results in particular [30, 33] (as providers appear to be influenced by their perception of patients’ expectations/wants in cash and kind). Also, the scaling up of MRDT for universal parasitological testing requires behavioural change interventions targeted at healthcare providers [9, 33], patients, families and community members [9]. However, there is a paucity of literature on interventional studies to increase patients’ and caregivers’ demands for MRDT the world over. Most studies have focused on the diagnostic accuracy of MRDT, uptake and use of MRDT among providers, appropriate response to negative test results, improvement in malaria treatment according to treatment guidelines and the cost-effectiveness of MRDT compared to presumptive and/or microscopic diagnosis, etc. This study aims to educate/sensitize social groups about MRDT and to train healthcare providers in health communication about MRDT with patients/caregivers, to determine whether these can increase the demand (use and/or request) for MRDT among community members in Ebonyi state, Nigeria, who have fever or malaria-like illness. The findings will inform health policy decisions in Ebonyi state and in Nigeria as a whole in her strive to achieve universal access to parasitological diagnosis of malaria together with the global communities.

## Methods/design

The study is a pragmatic, single-centre, three-arm, parallel, open-label, stratified cluster-randomized controlled trial with 1:1:1 allocation. “Pragmatic” means the study (interventions) would occur in natural settings or real-life situations. A cluster is defined as a geographical community or village(s)/settlement(s) (with at least 250 households or a population of 1500 people) serving as the proximate catchment area for at least one public primary health facility and one PMV within the study sites/strata in Ebonyi state.

A three-arm parallel design, with an equal number of clusters in each arm and an equal sample size in each cluster, will be used to assess the effect of two interventions compared to control:

1. Control arm**:** no intervention. This arm involves the usual practice of provision of MRDT services by individual healthcare providers (in public health facilities and PMVs) with basic training in MRDT. The PMVs that have offered the MRDT service previously but are not currently doing so will be re-supplied with MRDT kits to resume provision of MRDT services for the study period.
2. Social group arm: social group intervention. This arm involves control treatment plus the sensitization and education of social groups about MRDT.
3. Social group/provider arm: social group/provider intervention. This arm involves control treatment and the sensitization and education of social groups about MRDT plus the training of healthcare providers in health communication about MRDT with patients/caregivers (clients).

The trial protocol development was guided by the Standard Protocol Items: Recommendations for Interventional Trials (SPIRIT) 2013 checklist (see Additional file 1).

### Study area

The study is being conducted in three sites/strata in Ebonyi state, south-eastern Nigeria. Ebonyi state is located in the south-east geopolitical zone of Nigeria between latitude 5^0^ 40′ and 6^0^ 54′ N and longitude 7^0^ 30′ and 8^0^ 30′ E, with a land area of 5953 km^2^ [34]. It shares borders with Benue state to the north, Enugu state to the west, Cross River state to the east and Imo and Abia states to the south [34, 35]. The State lies in the plains of Cross River, with the rainy/wet season from April to October and dry/*harmattan* season from October to March [34]. Floods often occur during the rainy season due to poor drainage systems, stagnant rivers and ponds, which expose the State to mosquito infestations and a high malaria burden [34]. The prevalence of malaria in the State among children under 5 years of age as reported in the 2015 NMIS was 51% (diagnosed using MRDT) and 30% (diagnosed using microscopy) [15]. A total of 305,879 laboratory-confirmed cases of malaria were reported between December 2013 and November 2014 in the State’s malaria surveillance system, which only covers the public health sector [36]. The vegetation of the State is mostly savannah in the drier northern part and forests in the wetter southern part [34].

The State’s projected population for 2016 was 2,897,401 (based on the 2006 national census figure and a growth rate of 2.8%) with male inhabitants making up 48.62%, female inhabitants 51.38% and children under 5 years of age 20% [34]. The people of Ebonyi are primarily of the Igbo language and ethnic extraction with ten dialects/minor languages spoken across the State [35]. English, especially its local variant, the pidgin, is a widely spoken language in the State. People of other languages and ethnic groups in Nigeria also live in the State, especially in the capital and urban areas. Most inhabitants practice Christianity [35]. The State is divided into three senatorial zones (Ebonyi north, Ebonyi central and Ebonyi south), with 13 Local Government Areas (LGAs) (with Abakaliki LGA as the administrative and political capital), 64 development centres, 138 autonomous communities and 215 political wards [34]. Each autonomous community has a traditional ruler and consists of autonomous villages each having a village head called the chairman. Each autonomous village is made up of smaller villages or settlements each having a village/settlement head.

The public sector is the main driver of the State economy and agriculture is the major occupation [35]. The State has several solid mineral resources (including lead), crude oil and natural gas [35] but there are few large-scale commercial mines and industries [34, 35]. The State is called “the salt of the nation” because of huge salt deposit at the Okposi and Uburu Salt Lakes [35]. Traditional industries and works of art include blacksmithing, pottery works and wood works (carved doors, stools, walking sticks, traditional flutes, wooden mortars and pestles) [35].

Health care in Ebonyi state (like other states in Nigeria) is provided by the public and private sectors under the overall guidance of the federal government through the Federal Ministry of Health (FMoH) (and its agencies) and the national council on health. The federal government (through the FMoH) provides health services in the State through tertiary health facilities. The State government (through the State Ministry of Health (SMoH)) provides health care through secondary health facilities (general hospitals). The State government also supports the local governments in providing primary health care (PHC) through PHC facilities. The Ebonyi State Malaria Elimination Program (SMEP), within the department of public health in the SMoH, coordinates the efforts to combat malaria in the State.

Other healthcare service providers recognized by the National Health Act 2014 include the private healthcare providers, traditional healthcare providers and alternative healthcare providers [37]. These can be grouped as the private health sector and subdivided as formal private sector (private hospitals and clinics) and informal private sector (pharmacies, PMVs, traditional healers and alternative healthcare providers) [4]. The majority of febrile patients, especially from the lower socio-economic groups, seek care primarily in the informal private sector [14, 16, 18].

The three study sites/strata consist of the three senatorial zones: Ebonyi north, Ebonyi central and Ebonyi south. Ebonyi north consists of four LGAs with a 2016 population of 998,473 [38]. Two of these LGAs, Abakaliki LGA (the state capital) and Ebonyi LGA, have contiguous urban areas that jointly constitute the city/metropolis of Ebonyi state. However, Ebonyi north is a predominantly rural area. The vegetation is mostly savannah and it is drier than the other sites. Ebonyi central consists of four LGAs with a 2016 population of 861,554 [38] and is a predominantly rural area. The vegetation changes from savannah towards forests and the weather become wetter as one moves through this central zone southward towards the southern zone. Ebonyi south consists of five LGAs with a 2016 population of 1,037,376 [38] and is also predominantly rural. Malaria is endemic in the three sites, with year-round transmission and a high burden of malaria.

### Participants and participant timeline

#### Participants

##### Clusters

Clusters eligible to participate in the study include villages/settlements (a community) that have at least one eligible public primary healthcare facility and one eligible PMV (see below) and are easily accessible (close to a motorable road) even in the rainy season. Clusters either participating in or that have participated in any similar interventions within the preceding year will be excluded from participating in the study. Clusters that are too close (less than 15 km apart) and are not separated by a buffer area or natural barrier, and urban clusters (in cities/towns) will be excluded from participating in the study to minimize contamination between clusters within strata [39, 40]. Non-consenting clusters will also be excluded from participating in the study.

##### Social groups

Social groups are any set of persons within society with particular demographic, economic or social characteristics [41]. Examples of social groups include women associations/meetings, village meetings, men associations, youth associations, market/trade unions, elders’ fora, parent-teacher associations, etc. Social groups that are recognized by cluster heads/authorities will be included to participate in the study and non-consenting social groups will be excluded.

##### Primary health facilities and individual healthcare providers

Eligible public primary health facilities are those providing MRDT services, maternal and child healthcare services including immunization, attending to at least an average of four patients with fever (or suspected malaria) per day and having at least two staff that are at least junior community health extension workers (JCHEWs). Individual public healthcare providers involved in the diagnosis (and treatment) of malaria in these facilities are eligible. Eligible PMVs are those with basic training in MRDT services and either currently offering or having previously offered MRDT services. Non-consenting health facilities and individual providers will be excluded from the study.

##### Households and community members

Households will be eligible to participate in a population-based household survey if there is a report of any case of fever or suspected malaria among children under 5 years of age, children age 5 years and above and adults (excluding pregnant women) in the household in the 2 weeks preceding the survey. Consent will be obtained in each household, from the mother of the house or the female primary care-giver, who will be the respondent in the survey. Non-consenting households will be excluded from the survey. After the baseline household survey, community members that have resided in the community for at least 2 years will be eligible to participate in a focus group discussion (FGD). Non-consenting community members will be excluded from the FGD.

##### Participant timeline

**Fig. 1:**
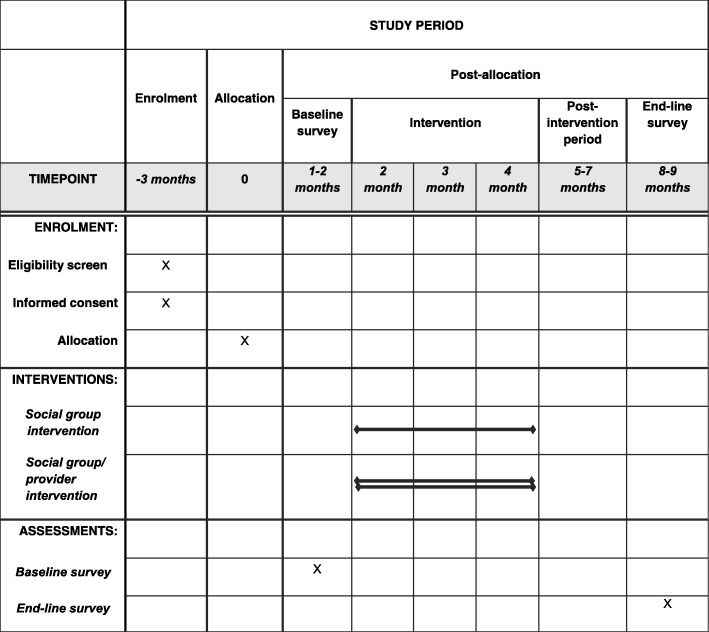
Schedule of enrolment, interventions and assessments

Informed consent to participate in the study will be obtained from the selected eligible participants (clusters, social groups and providers) before clusters are randomly allocated into the three study arms. The main study outcomes will then be measured through a baseline household survey and an end-of-study household survey 3 months after the end of the intervention. The participant timeline is depicted in accordance with the Standard Protocol Items: Recommendations for Interventional Trials (SPIRIT) guideline (see Fig. 1).

### Interventions

The interventions in this study include (1) the sensitization and education of social groups about MRDT (social group intervention) and (2) the sensitization and education of social groups plus the training of healthcare providers in health communication about MRDT with clients (social group/provider intervention). Participants in the control arm will not receive any of these interventions.

### The sensitization and education of social groups

The social group intervention will involve the sensitization and education of social groups within each cluster in the social group arm. There will be three episodes of group discussion/interaction, one per month, with each social group during the intervention period of 3 months. Health education messages designed to change incorrect beliefs and perceptions, promote correct beliefs and perceptions, increase knowledge and promote key actions will be communicated to social group members in an interactive session/group discussion. The health education message is adopted from a national framework [42] and modified for the study.

Each discussion will be a 1-hour event. One investigator will moderate each discussion with a group discussion guide. In order to make the intervention more pragmatic and to optimize adherence to the trial intervention, the discussions will take place at the usual meeting time of each social group, or at an agreed time, and at their usual meeting point/venue or at a central location as is most convenient for the participants. The intervention will be discontinued for a social group or cluster whenever such a request is made by the respective social group head/leader or cluster head/authority. The first episode will be divided into 7 sub-headings, viz. (1) introduction, (2) individual beliefs and opinions, (3) key facts about malaria, (4) key facts about MRDT, (5) demonstration of testing with MRDT kit, (6) key actions to practice and promote and (7) closing remarks. The moderator will explain the research problem and objectives to the participants and the broad role the participants (social group members) are expected to play in addressing the problem. Each participant will then share his/her beliefs and opinion about malaria with respect to the cause, symptoms, complications/fatalities, diagnosis, treatment, where to seek care and why and prevention. The discussion will explore the reasons for and against these views and there will be questions and answers from participants. The moderator will then highlight the key facts about malaria. Key facts about the MRDT will also be highlighted and discussed. Malaria rapid diagnostic test (MRDT) will be demonstrated in volunteer participants, the procedure will be explained, the results interpreted and explained to them and those that test positive will be treated with an ACT.

The moderator will then highlight and discuss the key actions that the participants should exercise and promote as individuals, social group members, opinion leaders, parents, heads of households, family members, friends and relatives, neighbours, etc. These actions include recognizing the symptoms of malaria especially in children under 5 years of age, promptly seeking care with providers that carry out MRDT (preferably in the public primary health facilities) and requesting the “simple test” for malaria (MRDT), receiving the test, asking for the result, and receiving treatment for malaria (with ACTs) only when the test is positive, making sure children under 5 years of age receive treatment within 24 hours of on-set of fever and acquiring and giving the appropriate ACTs at the correct dose for the correct number of days. The moderator will then make closing remarks and thank the participants for their time.

The second and third episodes will take a similar format as the first but will focus more on re-emphasizing the key facts and actions to practice and promote so as to reinforce participants’ knowledge and attitudes. The participants will be encouraged to share their experiences in the preceding 4 weeks for discussion and clarification. Visits to the social groups will end with the third episode of group discussion. However, group members will be able to contact the investigators on their cell phones if they still require clarification on malaria and MRDT during the subsequent months.

Other supportive interventions will include reminder text messages sent at least weekly to social group members that have cell phones, regular phone calls to social group heads (to urge them to remind and encourage the group members during meeting sessions) and regular visits by a provider (PMV or public) to the scheduled meetings of each social group for provision of MRDT services and ACTs for positive results.

### The training of healthcare providers

The clusters in the social group/provider arm will also receive the social group treatment as described above and in addition, will receive provider training in health communication about MRDT with clients. Health communication involves the use of communication strategies to inform and influence individual and community decisions that enhance health and it is a vital aspect of provider-patient relations [43]. The provider training will involve a one-day sensitization and training workshop for the participating healthcare providers in Abakaliki (the capital of Ebonyi state). The research team will administer the workshop with the aid of a PowerPoint slide presentation, a provider training guide and a health communication guide. There will be a pre-post-test to assess the knowledge of participants about malaria, MRDT and communication. The training will be divided into two parts with 4 sessions in each. The first part will focus on sensitizing the participants. The 4 sessions include (1) pre-test, (2) background information on the research problem and aim (objectives) and on malaria, (3) demonstration of testing using the MRDT kit and (4) key actions to practice and promote. The facilitators of the training will use a PowerPoint slide presentation to highlight the objectives of this part of the workshop and then to present an explanation of the problem the research is designed to address and how, the research objectives and the broad role the participants/providers are expected to play in this respect. Volunteer participants will then perform and receive MRDT, explain the procedure and interpret the results. This will be followed by a question and answer session on the sensitivity and specificity of MRDT, adherence to test results and what to do if result is negative and the benefits of universal testing and complete adherence to the test results. The facilitators will then highlight the key actions that need to be practiced and promoted by providers. Questions and comments will be respectively answered and discussed.

The second part of the training will focus on improving the health communication skills of participants with the aid of a health communication guide. The 4 sessions in this part include (1) background information on health communication, (2) a practical session (including simulation) on health communication with clients, (3) a closing session and completion of assessment forms and (4) post-test. The facilitators will use a PowerPoint slide presentation to highlight the objectives of this part of the workshop and to talk about communication and health communication with regards to definitions, communication components, process, channels/media, types, aids, etc. This will be followed by a practical session and simulation of a real-life situation. The facilitators will then divide the participants into two groups – the public provider group and the private provider group. Each group will be sub-divided into clients and providers. Each client/patient sub-group will visit their respective providers with malaria-like illness. Their providers will then engage them in health communication about malaria and MRDT (with or without the use of a self-written/improvised guide). The strengths and weaknesses of each group’s communication techniques during the simulation will be noted by the other group. These will be read out by a facilitator for discussion by all parties.

The facilitators will then introduce the participants to a health communication guide on how to effectively educate their clients during the discharge of their duties. They will be educated on how to use the guide in communicating with all categories of clients including those that requested MRDT or freely accepted providers’ suggestion of a test or initially declined to receive the test. The participants will then apply the guide in another round of simulation exercise. Participants will then complete an assessment form on which they will rate the training process and logistics, state what they believed or knew previously that has now been corroborated, what they believed or knew previously that has now been contradicted, what they just learnt for the first time and any additional key actions that can be taken or promoted to increase the use of MRDT and adherence to test results. The participants will be encouraged to use the health communication guide to effectively communicate with their clients (suspected malaria cases) about MRDT at the course of their regular duties. A facilitator will then give the closing remark and implore the participants to develop their communication skills by putting it to practice immediately.

The training workshop will be followed by a twice weekly reminder text message and a monthly visit to each of the participating providers for supportive supervision (on-the-job training) and monitoring throughout the rest of the 3-month intervention period. Each support visit will be aimed at assessing providers’ performance, exploring their experiences in the previous weeks and addressing their challenges in health communication with their clients. The on-the-job training will make the intervention more pragmatic and will optimize adherence to the trial intervention. The healthcare provider’s intervention will be discontinued whenever requested by the respective provider.

The trained providers will also be subjected to mystery client monitoring. The mystery client assessment of providers’ performance will guide the researchers in properly identifying those that require more attention, and in what specific areas, during the support visits. Mystery clients will be trained on how to interact with providers and how to complete the assessment form thereafter. A mystery client will visit a participating provider/health facility and claim he has malaria based on familiar symptoms. If proposed by the provider, he/she will freely accept MRDT, otherwise, he/she will request MRDT. He/she will all the while note whether the provider engaged him/her in health communication with basic standard techniques. He/she will then complete the assessment form after leaving the provider. Each provider/health facility will be visited by a mystery client at least twice.

### Objectives

The primary objectives include:

1. To evaluate the effectiveness of the sensitization/education of social groups about MRDT (social group intervention) in increasing the demand (use and/or request) for MRDT compared to usual practice (control)
2. To evaluate the effectiveness of the sensitization/education of social groups about MRDT and training of providers in health communication about MRDT with patients/caregivers (social group/provider intervention) in increasing the demand for MRDT compared to usual practice (control)
3. To evaluate the effectiveness of the social group/provider intervention in increasing the demand for MRDT compared to the social group intervention

The secondary objectives include:

1. To evaluate the effect of the interventions on the care-seeking practices and pattern of anti-malarial drug use among community members
2. To assess the effect of the interventions on the knowledge and opinions of respondent female heads of households (female primary care givers) about malaria and malaria diagnosis
3. To evaluate the effect of the interventions on the number of patients with suspected malaria visiting the public primary health facilities
4. To assess the effect of the interventions on the knowledge and opinions of healthcare providers and their practice of health communication about malaria and malaria diagnosis
5. To evaluate the cost-effectiveness of the interventions
6. To assess the level of demand (use and/or request) for MRDT, care-seeking practices and pattern of anti-malarial drug use among community members with fever or malaria-like illness
7. To assess the level of knowledge and opinions of female heads of households (female primary care givers) and of healthcare providers about malaria and malaria diagnosis
8. To ascertain the factors that influence the demand for MRDT among community members

### Hypotheses

1. The social group intervention is more effective (and more cost-effective) in increasing the demand (use and/or request) for MRDT compared to control.
2. The social group/provider intervention is more effective (and more cost-effective) in increasing the demand for MRDT compared to control and compared to social group intervention alone.

**Fig. 2:**
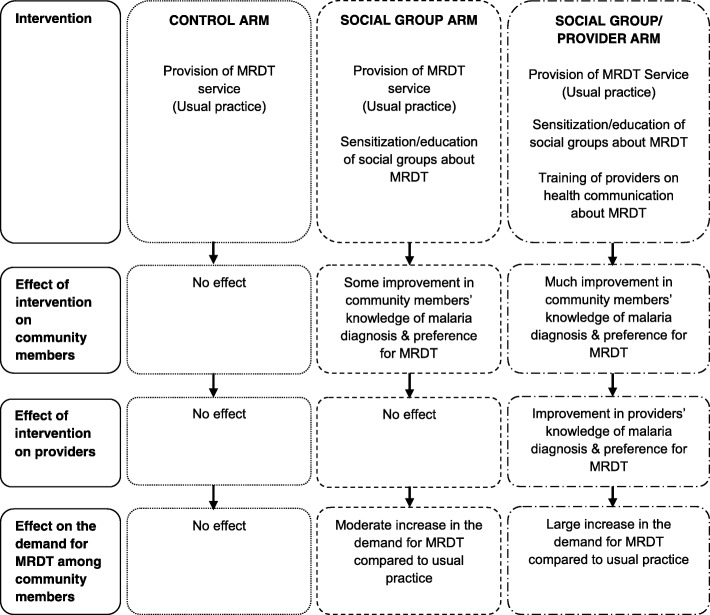
Summary of the study’s logical framework

The social group intervention is expected to enhance the knowledge and opinions of community members about malaria and malaria diagnosis and their preference for MRDT. This will lead to a moderate increase in their demand for MRDT compared to community members in the control arm. The social groups/provider intervention is expected (to a greater extent) to enhance the knowledge and opinions of the community members and their preference for MRDT. This intervention will also enhance the knowledge and opinions of healthcare providers about malaria and malaria diagnosis and their preference for MRDT. These will lead to a large increase in the demand for MRDT among community members compared to community members in the control arm. The summary of the study’s logical framework is shown in Fig. 2.

### Outcome measures

#### Primary outcome

1. The primary outcome is the proportion of children under 5 years of age with fever or malaria-like illness, in the 2 weeks preceding a population-based household survey, who received MRDT
2. The co-primary outcome is the proportion of children ages 5 years and above and adults (excluding pregnant women) with fever or malaria-like illness, in the 2 weeks preceding a population-based household survey, who received MRDT

#### Secondary outcomes

The main secondary outcomes at the community level include:

1. The proportion of these children under 5 years of age, who received MRDT, whose caregivers requested the MRDT
2. The proportion of these children ages 5 years and above and adults, who received MRDT, who requested or whose caregivers requested the MRDT
3. The proportion of children under 5 years of age with fever or malaria-like illness, in the 2 weeks preceding a population-based household survey, whose caregivers sought care with a provider, those that sought care the same or next day and among those for whom care was sought, the type of provider with whom care was sought
4. The proportion of these children under 5 years of age who took any anti-malarial drug and who took ACTs.
5. The proportion of children ages 5 years and above and adults (excluding pregnant women) with fever or malaria-like illness, in the 2 weeks preceding a population-based household survey, who sought care with a provider, those who sought care the same or next day and among those who sought care, the type of provider with whom care was sought
6. The proportion of these children and adults who took any anti-malarial drug and who took ACTs.
7. The proportion of respondent female heads of households (female primary care givers) with good knowledge and opinion about malaria and malaria diagnosis.

The secondary outcomes at the provider level include:

1. The number of patients with suspected malaria visiting the public primary health facilities (monthly) as identified in the patients’ register
2. The proportion of providers with good knowledge and opinion and good health communication about malaria and malaria diagnosis.

Cost outcomes include the total cost of the social group and social group/provider interventions, average cost per provider who participated in the provider training and average cost per social group member who participated in the social group sensitization/education.

### Measurement of study outcomes

#### Population-based household survey

The survey questionnaire was adopted from the 2015 NMIS [15] woman’s questionnaire and was modified to collect data on both the primary outcome and community-level secondary outcomes. The questionnaire will be pre-tested in non-participating clusters before the survey. Interviewers will be recruited and trained over a 1-week period to administer the questionnaire. The training of the interviewers will include a detailed review and explanation of the questionnaire items, interview techniques, how to provide information to household respondents about the survey, how to obtain consent, the translation of key words in the questionnaire to local language and how to administer the questionnaire. The baseline survey will be carried out before the intervention (see Fig. 1) to collect data for the assessment of the level of demand (use and/or request) for MRDT among community members with fever or malaria-like illness in Ebonyi state. A section of the questionnaire will collect data on the determinants of the demand for MRDT. The end-of-study survey will be carried out 3 months after the end of the intervention (see Fig. 1) to collect data for the evaluation of the effects of the interventions on the demand for MRDT among community members.

The interviewers will be accompanied by research supervisors at the start of the baseline survey for monitoring and supportive supervision. This will be followed by a visit at least once weekly for monitoring and supportive supervision. The household survey questionnaire is designed to collect data about the following items from the respondent female head of household (the mother of the house or female primary care giver):

1. Basic socio-demographic characteristics (of the respondent and of eligible household members reported to have had fever/malaria-like illness in the preceding 2 weeks)
2. Fever/malaria-like illness management including care seeking and demand for MRDT
3. The respondent’s knowledge and opinions about malaria and malaria diagnosis
4. The determinants of the demand for MRDT

#### Focus group discussion (FGD)

The FGDs will be conducted at baseline to collect data on the factors that influence the demand (use and or request) for MRDT in the communities. Nine focus group discussions (FGDs) will be carried out across the three study sites/strata. Participants will be purposively selected from among providers, male and female community members (who have resided in the community for at least 2 years). There will be three focus group discussions (FGDs) with providers, one per stratum; three FGDs with male community members, one per stratum and three FGDs with female community members, one per stratum.

Investigators will administer the FGDs using a FGD question guide prepared in English and pre-tested in non-participating clusters. The FGD question guide will consist of both very open-ended and more targeted questions designed to explore the providers’ and community members’ knowledge and practice of MRDT and their perceptions of the determinants of the demand for MRDT in their communities/villages. The more targeted focus group questions are based on the proximal determinants in the study’s logical framework. The more targeted questions are combined with the very open-ended questions to identify additional determinants. Before commencement of each FGD, the investigators will collect background data on participants such as age, sex, level of education, occupation and number of years of experience in using MRDT (for providers). Each FGD will consist of 8–10 participants and will last for about 1 hour. The FGD will be audio-recorded and later transcribed (and translated) verbatim into English before analysis.

#### Provider survey

Provider survey will be conducted at baseline and end of study (3 months after the end of the intervention) to collect data for the assessment of the effect of the interventions on the knowledge and opinions of healthcare providers and their practice of health communication about malaria and malaria diagnosis. The provider survey questionnaire is similar to the household questionnaire and is modified to collect data about the following items from the healthcare providers:

1. Basic socio-demographic characteristics
2. Knowledge and opinions about malaria and malaria diagnosis
3. The practice of health communication about malaria and malaria diagnosis
4. The determinants of the demand for MRDT

#### Review of the patient register and personal medical records

Patient registers are routinely kept by public primary health facilities. The following relevant data are contained in the registers: the patient’s biodata (name, age, sex, address, etc.), date, symptoms, type of test performed, test result and type of drug given. The participating PMVs will be asked to keep similar records. The registers will be used by the research team for monitoring, supportive supervision and evaluation. The investigators will review the patient registers for 9 months pre-intervention and 9 months post end-of-intervention records. The document review will provide data for evaluating the effect of the interventions on the number of patients with suspected malaria visiting the public primary health centres.

#### Documentations of the implementation/intervention process

The process of implementation of the study will be documented including challenges encountered and how these were addressed. The social group and provider intervention processes will be documented and/or recorded with respect to the name of the participant, the participant’s phone number/contact, individual contributions, the participant’s observations and evaluation of the intervention logistics and suggestions, etc. The study personnel and the organizers of the interventions will also record their experience on challenges and possible solutions. These data will be used for monitoring and to guide the implementation of similar interventions in the future.

#### Costing of the intervention process

Direct and indirect costs of the intervention process will be assessed from the perspective of the implementer of the intervention and that of the recipient of the intervention (the societal perspective) using standard economic evaluation methods. Costs will be estimated primarily from the documentation of the implementation/intervention process and the project financial accounts. The costing will be guided by the research budget.

#### Data management plan and quality assurance

During the household surveys, the research supervisors will revisit an average of 30 households in each participating cluster with a specially designed questionnaire to double check on responses and coverage. The supervisors will collect completed questionnaires from the interviewers weekly and crosscheck/review for internal consistency and completeness. Questionnaires with internal inconsistencies and/or missing data will be returned to the respective field staff for correction with the respective respondent. The questionnaires will be serially (and uniquely) numbered and data will be double-entered using Microsoft Excel 2007 (Microsoft Inc., Redmond, WA, USA) and will be verified using Stata version 15 (Stata Corp, College Station, TX, USA). The dataset-compare utility in Stata will be used to verify the datasets and any discrepancies identified will be crosschecked against the corresponding original questionnaire and corrected before analysis. The variables and data in the dataset will also be examined in detail and range checks will be done to ensure the data were entered correctly and appropriately.

The audio recordings of the FGDs will be transcribed (verbatim) within 24 hours of recording and translated into English before analysis. The questionnaires, the audio recordings and the verbatim transcript of the FGDs will be stored in a secure area while the electronic data file will have a back-up file. Access to study materials and data files by unauthorized persons will be prevented.

#### Sample size

After comparing the required sample size for each of the primary objectives, the largest sample size will be used. This is based on the primary objective to evaluate the effectiveness of the social group/provider intervention compared to the social group intervention in increasing the demand for MRDT in the communities. The sample size is estimated using the methods recommended for a stratified, cluster-randomized trial [39, 40] and will be based on the primary outcome of the proportion of children under 5 years of age with fever or malaria-like illness, in the 2 weeks preceding a population-based household survey, who received MRDT. A plausible estimate of the primary outcome of 11% in the control arm will be used, based on the reported value for the south-east zone (that includes Ebonyi state) in the NMIS 2015 [15].

Assuming a coefficient of variation between clusters within strata of 0.16, a sample size per cluster of 40 eligible children under 5 years of age and 80% power at 5% probability of type I error, the trial will require 6 clusters per arm to detect a difference of 20% in the primary outcome between the control arm and the social group arm (an increase from 11% to 31%) and a difference of 20% between the social group arm and the social group/provider arm (an increase from 31% to 51%). A sample size per cluster (cluster size) of 40 subjects with 6 clusters per arm gives a total cluster number of 18, with 240 subjects per arm and a total sample size of 720 subjects. The sample size per cluster (of 40) will be increased by 20% (to 50) to compensate for any probable invalid records and other field eventualities. This gives a final cluster size of 50 subjects with 300 subjects per arm and a total sample size of 900 subjects.

The same sample size will be used to assess the co-primary outcome of the proportion of children ages 5 years and above and adults (excluding pregnant women) with fever or malaria-like illness, in the 2 weeks preceding a population-based household survey, who received MRDT. An equal number of clusters (*n* = 6) will be allocated to each arm and to each stratum and, despite variations in population size in the clusters, an equal fixed sample size (*n* = 50 for each target population) per cluster will be used to optimize statistical efficiency [39, 40].

Sample size is estimated for the provider survey to give the expected level of precision (at a 95% confidence level) for determining the proportion of providers that have good knowledge and opinion about malaria and malaria diagnosis. Based on the number of clusters (*n* = 6) allocated to each arm and about 7–8 individual providers per cluster who consented to participate in the study, we expect to survey 42 providers per arm (7 per cluster). Assuming the outcome measure of 50% in each of the control and social group arms and 70% in the social group/provider arm, with an intra-cluster correlation coefficient (ICC) of 0.01, we can estimate the true outcome measure with ± 15.6% precision in each of the control and social group arms and ± 14.3% precision in the social group/provider arm.

#### Sampling technique (recruitment)

**Fig. 3:**
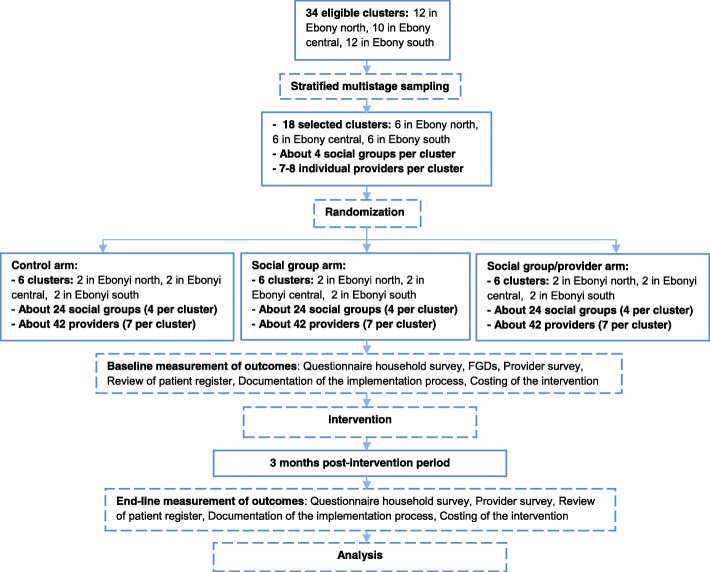
Summary of the trial profile

Stratified multistage (cluster) sampling will be employed. We will randomly select 18 clusters from a list of eligible clusters across 3 strata (6 in each stratum) using the “sample” command in Stata. To enhance reproducibility, random-number seed will be set using the “set seed” command before running the “sample” command. If written consent is not provided by any of the selected cluster(s) before randomization, replacement cluster(s) will be randomly selected from the remaining list of eligible clusters using the same technique. The summary of the trial profile is shown in Fig. 3.

Within the selected clusters, an average of four eligible social groups per cluster will be purposively selected and non-consenting social group(s) prior to randomization will be replaced. All eligible and consenting health facilities and the individual providers involved in the diagnosis and treatment of suspected cases of malaria will be selected across clusters before randomization. A number of eligible individual providers and community members who have resided in the community for at least 2 years will be purposively selected for the focus group discussion. All the households in the selected clusters will be enumerated and screened for inclusion during the pre-intervention and post-intervention surveys. If the required sample size is not reached after the interviewers have reached the end of the enumerated households, non-eligible households will be revisited and re-assessed to see whether they have become eligible.

#### Randomization

Randomization will be done using Stata version 12 (Stata Corp, College Station, TX, USA). A statistician that will not otherwise take part in the study will use stratified randomization to assign the 18 clusters across the 3 strata to the 3 treatment arms after written consent is obtained from all recruited participants. The six clusters within each stratum will be randomly allocated to the three study arms in the ratio of 2:2:2 using a programme written in Stata. A restricted randomization technique will be employed for the within-strata allocation, to ensure the desired balance in cluster number in the study arms. The summary of the trial profile is shown in Fig. 3.

It will not be possible to blind the interviewers who administer questionnaires in the household survey or the respondent female heads of households, because of the pragmatic nature of the trial, as they could acquire treatment knowledge from informal sources or *on the grapevine*. Some of the respondents could also be social group members or providers. It will also not be possible to blind the investigators who administer the intervention or the participants receiving the intervention (social groups and providers).

#### Data analysis

Data will be double-entered using Microsoft Excel 2007 (Microsoft Inc., Redmond, WA, USA) and analysed using Stata version 15 (Stata Corp, College Station, TX, USA). Since the number of subjects/household members on whom data will be collected is expected to vary across clusters (due to the method employed for the household survey), the “sample” command will be used to randomly select an equal number of subjects across clusters for analysis. All analyses will be on an intention-to-treat basis.

The effect of the interventions on the primary outcome will be analysed using cluster-level methods for stratified, cluster-randomized trials with a small number of clusters per treatment arm [39, 40]. Point estimates of the intervention effects (risk difference) in each intervention arm compared to control and in both of the intervention arms compared to each other will be computed from the unweighted mean of cluster-level summaries (proportions) of the outcome measures in each study arm. But since the cluster size (sample size across clusters) is constant, this point estimate will be identical to that obtained from the weighted average of individual values [39, 40]. Also, since the number of clusters across strata is constant, the estimated risk difference will be identical to the weighted average of stratum-specific risk difference.

An overall test of the null hypothesis of no difference between any of the study arms will be conducted in a two-way analysis of variance (ANOVA) of the cluster-level proportion on stratum and treatment arm. This overall test is to guide the interpretation of any subsequent significant findings in pair-wise comparisons. If the distribution of the cluster summaries in each study arm is markedly skewed, logarithmic transformation may be considered before analysis. Before conducting pair-wise significance testing and computing a confidence interval, an estimate of the within-stratum between-cluster variance will be obtained as the residual mean square from two-way ANOVA of the cluster-level proportions on stratum and treatment arm, including interaction terms. The within-stratum between-cluster variance will then be used in a stratified *t* test to test the null hypotheses of no difference in the primary outcome between each intervention arm and the control and between the two intervention arms. It will also be used in computing the corresponding 95% confidence interval of each risk difference.

Adjusted analysis based on cluster summaries will be done in a two-staged procedure. In the first stage, the covariate-adjusted residual will be obtained for each cluster using standard multiple linear regression analysis, incorporating the stratum (as a fixed effect) and all baseline cluster-level covariates of interest but excluding the intervention effect. The potential baseline covariates of interest will include cluster-level summaries of the primary outcome measure, mean scores for respondents’ knowledge and opinions (about malaria and malaria diagnosis) and other baseline variables found to differ between the study arms and which can be determinants of a particular outcome. Only cluster-level summaries of baseline covariates will be used in the adjusted analysis because baseline and follow-up data will be on different individuals, as the study employed a repeated cross-sectional design [39, 40]. The difference-residual for each cluster will be obtained as the difference between the observed outcome in each cluster and the predicted outcome in the absence of an intervention effect. The covariate-adjusted residuals will replace the cluster-level proportions in the second stage in estimating the intervention effects, which are thus adjusted for the covariates in the first stage.

The same cluster-level methods will be used to evaluate the effect of the interventions on the secondary outcomes. Comparative analysis of baseline data will be used to assess the balance between the treatment arms and potential baseline variables that will be reported include the cluster-level and individual-level summaries of the primary and secondary outcome measures, the age and sex of individual subjects, the age of the respondent female head of household, educational level, occupation, level of knowledge and opinions about malaria and malaria diagnosis and average knowledge and opinion score. Summary statistics of baseline data will be used to assess the level of demand for MRDT, care-seeking practices and pattern of anti-malarial drug use among community members with fever or malaria-like illness; the level of knowledge and opinions of the female heads of households about malaria and malaria diagnosis and the level of knowledge and opinions of healthcare providers about malaria and malaria diagnosis.

To ascertain the factors that influence the demand for MRDT in the communities, a thematic analysis of the FGDs will be performed using the method recommended by Braun and Clarke [44]. The audio recordings of the FGDs will be transcribed (and translated) verbatim into English and the transcript will be compared with the original recording to check for “accuracy” before conducting the analysis. Exact and meaning-based translation will be used. QDA Miner Lite 2 (by Provalis Research) will be used to manage the coding and analysis process.

## Discussion

This study, in investigating whether the social group and social group/provider interventions will increase the demand for malaria rapid diagnostic test (MRDT) among community members, will use a cluster-randomized design due to its pragmatic nature, as it is occurring in natural settings, and to minimize contamination as the interventions are behavioural and are delivered to groups of individuals. The research team is collaborating with the Ebonyi State Malaria Eradication Programme (SMEP) to enhance successful implementation of the study. Continuous collaboration with the Ebonyi SMEP, and by extension the National Malaria Elimination Programme (NMEP), will provide an opportunity for the study findings to contribute to both state and national policies that will enhance the realisation of universal parasitological diagnostic testing in suspected cases of malaria. A potential limitation of this study is due to the fact that the study will, in most part, involve interviewing respondents about past behaviour and will thus be subject to recall bias. However, as the time period is short (2 weeks) the bias will be minimal. Any significant amendments to the trial protocol after protocol publication will be reported to the research and ethics committees and the registry body. Study results will be reported at local, national and international levels including in peer-reviewed journals and at national and international conferences.

### Trial status

The trial is ongoing. Recruitment of participants began on 27 August 2018 and is expected to end by 29 June 2019. Protocol version: original, dated 24 August 2018.

## Additional files

Additional file 1: SPIRIT 2013 checklist (DOC 122 kb)

Additional file 2: Ethical approval letter from the Research and Ethics Committee of the Federal Teaching Hospital Abakaliki (FETHA). (JPG 444 kb)

Additional file 3: Ethical approval letter from the Ethical Review Committee of the Ebonyi State Ministry of Health. (JPG 339 kb)

## Data Availability

The datasets used and/or analysed during the current study will be available from the corresponding author on reasonable request.

## Abbreviations

ACTs: Artemisinin-based combination therapies
ANOVA: Analysis of variance
FGD: Focus group discussion
MRDT: Malaria rapid diagnostic test
NMIS: Nigeria Malaria Indicator Survey
PMVs: Patent medicine vendors
WHO: World Health Organization
