# Supplementary figures and images for: Social group and health care provider interventions to increase the demand for malaria rapid diagnostic test among community members in Ebonyi state, Nigeria: study protocol for a cluster randomized controlled trial
Source: Trials. 2019 Oct 10;20:581. doi: 10.1186/s13063-019-3620-0 (PMC6785898; doi:10.1186/s13063-019-3620-0)

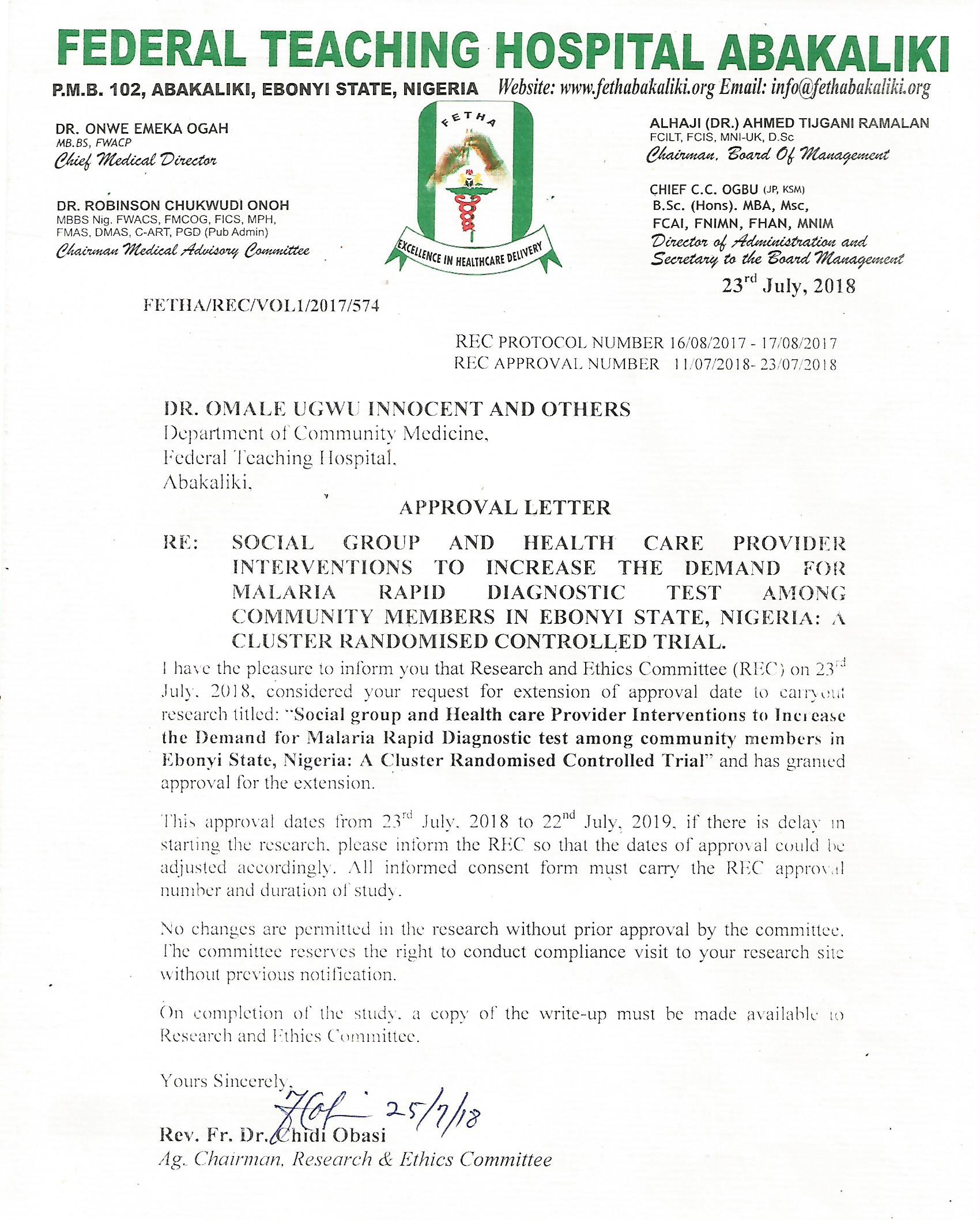

Supplement: Supplementary file 2 — Ethical approval letter from the Research and Ethics Committee of the Federal Teaching Hospital Abakaliki (FETHA). (JPG 444 kb) [file 13063_2019_3620_MOESM2_ESM.jpg]

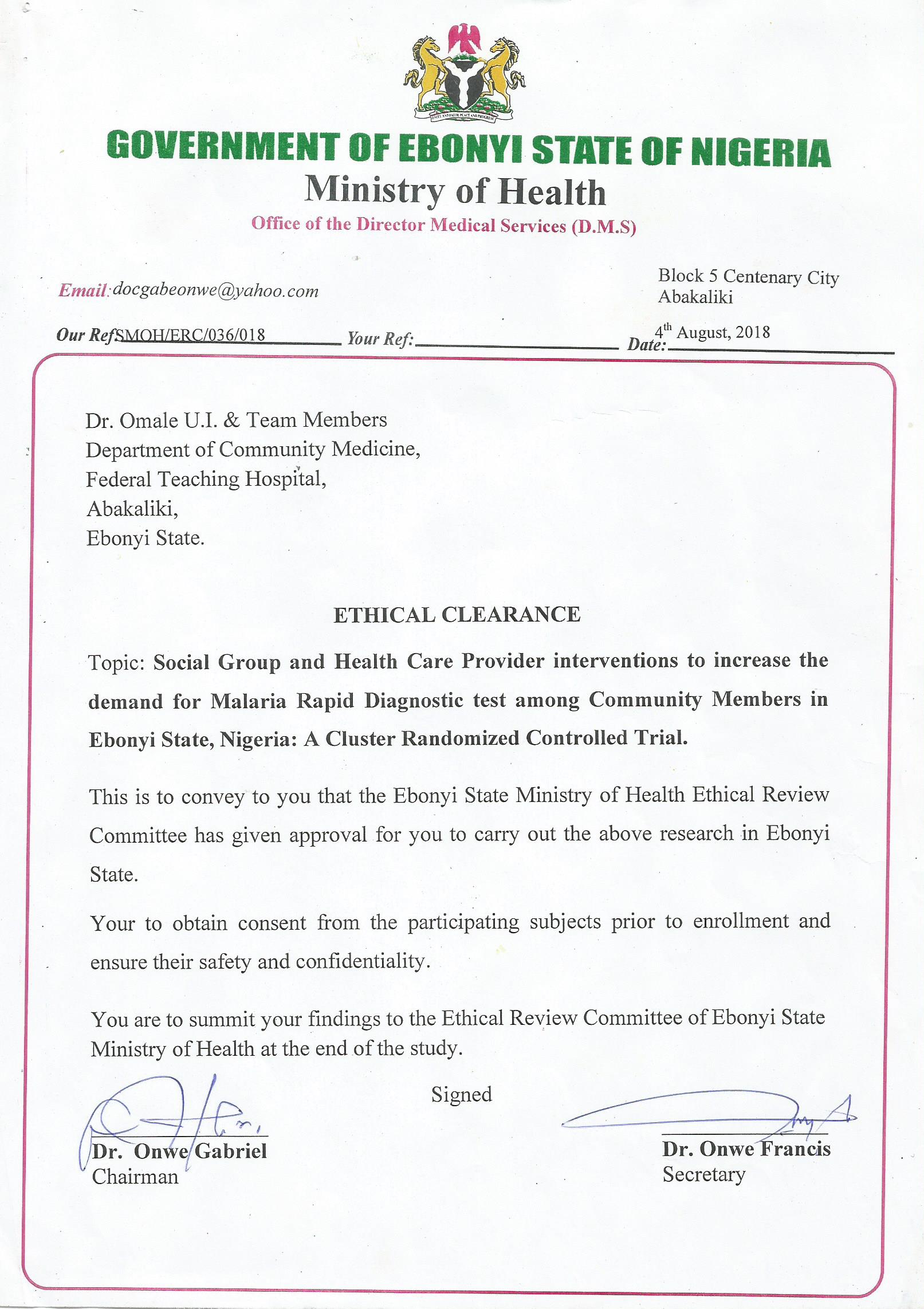

Supplement: Supplementary file 3 — Ethical approval letter from the Ethical Review Committee of the Ebonyi State Ministry of Health. (JPG 339 kb) [file 13063_2019_3620_MOESM3_ESM.jpg]
